# The Pathology of Hyperthyroidism

**DOI:** 10.3389/fendo.2018.00737

**Published:** 2018-12-03

**Authors:** Virginia A. LiVolsi, Zubair W. Baloch

**Affiliations:** Department of Pathology and Laboratory Medicine, University of Pennsylvania Medical Center, Philadelphia, PA, United States

**Keywords:** hyperthyroidism, thyrotoxicosis, non-hyperthyroid, Graves' disease, hyperfunctioning nodules, ectopic hyperthyroidism, drug reactions, mechanico-destructive

## Abstract

This article reviews those pathologic lesions which are associated with clinical and/or biochemical hyperthyroidism. Beginning with the descriptive pathology of classical Graves' disease and the less common toxic nodular goiter and hyper-functioning thyroid nodules, this paper describes the effects of non-thyroidal hormones, glandular function (including pituitary and hypothalamic lesions), ectopic production of thyroid stimulating proteins by non-thyroidal neoplasms, exogenous drug reactions causing hyper-function and finally conditions associated with a mechanic- destructive cause of hyperthyroidism.

## Introduction

Hyperthyroidism is a clinical syndrome characterized by hypermetabolic state due to the increased free serum thyroxine (T4) and/or free triiodothyronine (T3). There are many known factors and pathologies both inherent to the thyroid gland as well of non-thyroidal origin that lead to hyperthyroidism. It can result from hyperplasia and overstimulation of thyroid epithelium, acute destruction of thyroid follicles, and follicular epithelium due to various forms of thyroiditis or metastatic tumors. In addition, various drugs and antineoplastic agents can lead to thyroid dysfunction. In this review we provide a pathologist's perspective on various pathologic features that can be encountered in thyroids of patients with clinical hyperthyroidism.

## Toxic goiter

This condition can be divided into diffuse and nodular types.

### Diffuse toxic goiter

**Figure 1:**
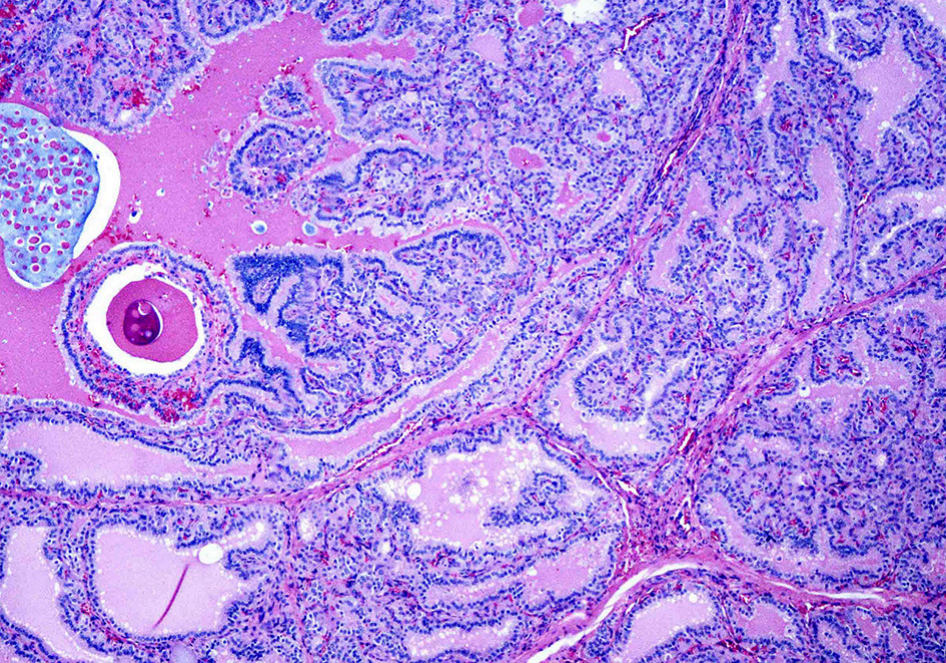
A case of Graves' disease on low power showing exuberant papillary hyperplasia.

**Figure 2:**
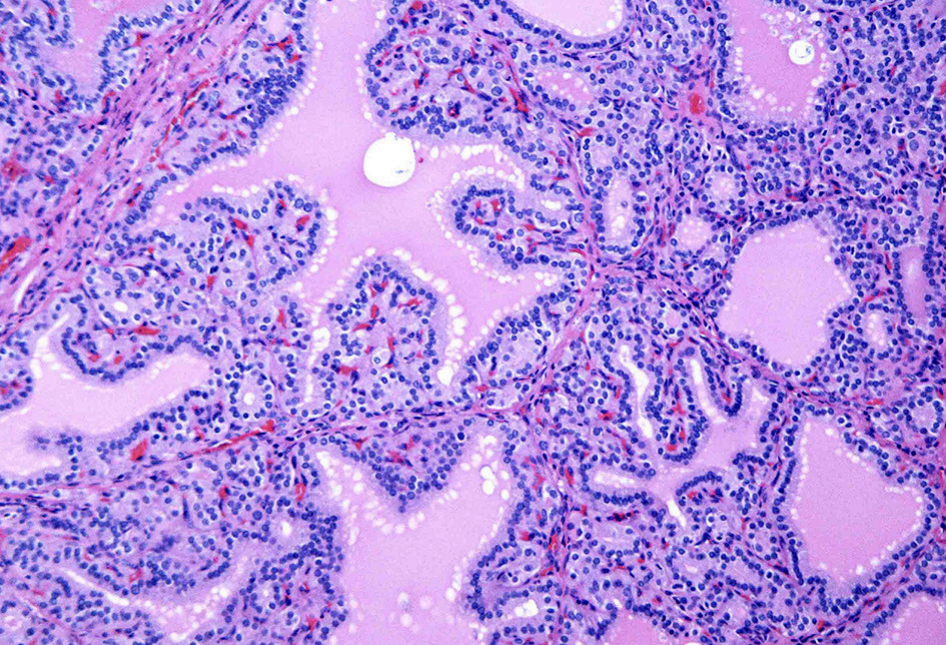
A case of Graves' disease on medium power showing cells with round nuclei and even chromatin pattern lining the papillae.

Most patients with classical hyperthyroidism caused by autoantibodies against the TSH receptors, stimulating thyroid follicular cell receptors show an enlarged hypervascular thyroid without obvious nodularity. This condition known in North America as Graves' disease and in Europe as Basedow disease is a disorder of young usually female patients who present with heat intolerance, tachycardia, tremors, weight loss and orbitopathy (1–6). This disease is characterized by thyroid enlargement with smooth capsule, non-nodular growth, and increased vascularity. Histologically one notes the presence of a diffuse papillary and follicular hyperplasia and varying degrees of lymphocytic infiltration into the thyroid stroma (7–9) (Figures 1, 2). In contrast to classic Hashimoto's disease, the lymphocytes do not infiltrate the follicular cells. The latter often are enlarged and can show cytoplasmic eosinophilia. The nuclei of these cells can also be enlarged and can in extreme cases mimic the nuclei of papillary thyroid carcinoma (10–12). However, the nuclei in Graves' disease tend to maintain a rounded shape and to have internal structure with minimal if any clearing (10–12).

There has been controversy regarding whether the presence of Graves' disease can lead to the development of papillary carcinoma and if the two coexist, does the carcinoma behave more aggressively than similar tumors not arising in this background setting. Several studies and our own experience have shown that the data needs to be evaluated systematically (13–17). If a papillary microcarcinoma is discovered incidentally in a surgically removed hyperthyroid gland, the prognosis is excellent. If a clinically evident tumor is identified in a Graves' patient, then the pathological characteristics of that lesion (size, extent, mutlifocality) will determine the prognosis. It does not appear that the background gland influences the prognosis adversely (18).

### Toxic nodular goiter

As the name implies is an enlarged gland with multiple nodules of varying sizes. Usually the nodules show the papillary and follicular hyperplasia although the lymphocytic stromal infiltration may be within the nodules and in the non-nodular thyroid (9, 19). The correlation between the histology of the nodules and radioactive iodide scan results is fair to poor since nodules with histologic evidence of hyperfunction often are warm or cool on scan. Toxic nodular goiter tends to occur in older individuals and affects males as well as females. In some older patients, the clinical manifestations of the hyperthyroidism may not be related to the thyroid at all; many of these individuals show symptoms related to cardiac disease, frequently atrial fibrillation. This disorder, sometimes referred to as “apathetic hyperthyroidism” needs to be considered by treating clinicians and appropriate laboratory testing will lead to the correct diagnosis (20–22).

## Hyperthyroidism associated with hyperfunctioning thyroid tumors

Most autonomously functioning thyroid tumors are benign that is follicular adenomas or hyperplastic nodules. These lesions are also designated as ‘autonomous nodules” and have been given the acronym “Plummer's disease” (23, 24).

Benign hyperfunctional adenomas (*AKA Toxic Adenoma*) are clonal, autonomously functioning follicular proliferations that produce supra-physiological amounts of thyroid hormones causing TSH suppression. These are more common in women and usually present at an older age. Usually, a radioisoptope scan confirms the preoperative diagnosis and most are not subjected to fine-needle aspiration (FNA). However, in rare cases a FNA is performed by a clinician or surgeon without the knowledge of thyroid function tests. In such cases, the FNA specimen is usually cellular and most likely will be diagnosed as a follicular neoplasm (Bethesda Category IV).

**Figure 3:**
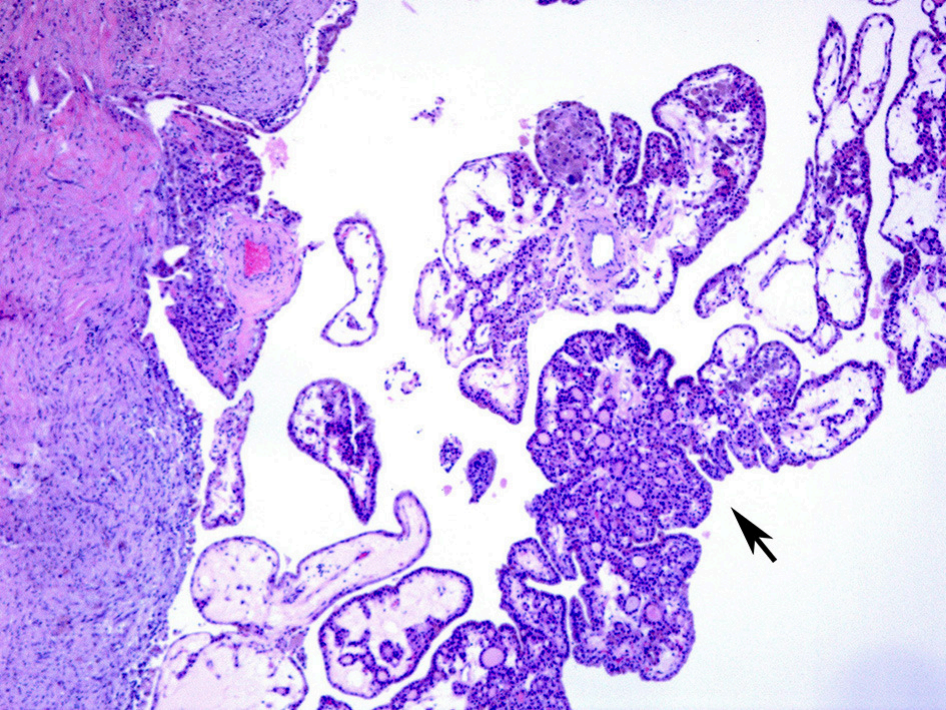
A case of papillary hyperplastic nodule on low power showing cystic nodule with papillary architecture (arrow).

**Figure 4:**
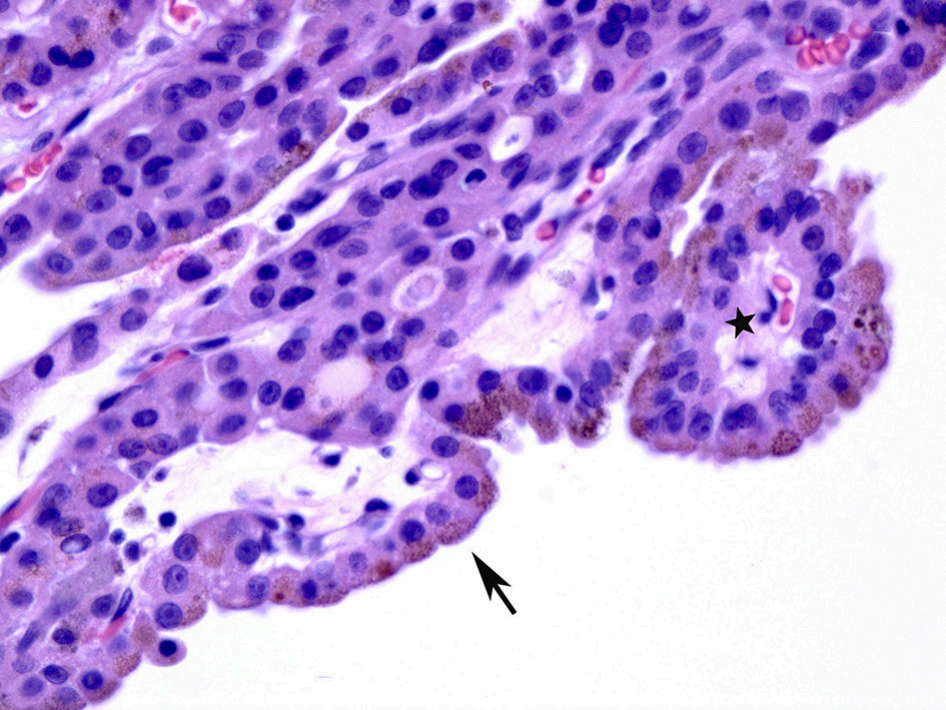
A case of papillary hyperplastic nodule on high power showing oncocytic cells lining the papillary structures (arrow).

On surgical excision the toxic adenoma grossly shows a distinct capsule and may be centrally cystic. These lesions can also show a papillary pattern of growth without nuclear features of papillary carcinoma. The autonomously functioning nodule usually occurs in young females. This lesion also termed “papillary hyperplastic nodule” (25) (a term coined by the late Dr. Austin Vickery) is an encapsulated or at least circumscribed area in the thyroid composed of exuberant papillary structures often with some follicle formation in the cores of the papillae; the lesions are often centrally cystic and the papillae tend to point toward the center of the nodule. Importantly the nuclei lining these papillary structures are round, have internal structure and are often polarized within the cells (Figures 3, 4). Lymphocytes are rarely found within these lesions (11, 12). Most of these nodules are clonal proliferations and are therefore considered adenomas (26–29). (The term “papillary adenoma” would be an appropriate one for these lesions; however, this term is shunned since it has been used to described encapsulated papillary carcinomas in older literature) (30). Although the great majority of these hyperplastic nodules are not associated with clinical hyperthyroidism, about 15–20% of affected patients do have symptomatic hyperfunction and about another 30% have biochemical hyperthyroid indices (25, 31, 32).

Rarely, malignant tumors of the thyroid may be associated with hyperthyroidism. These are usually but not always follicular carcinomas; some are encapsulated follicular variants of papillary carcinoma (33–35). Although the tumors may lead to hyperfunction while still confined within the gland, many of the affected patients have metastatic disease. Some authors indicate that tumor burden correlates with the degree of hyperthyroidism (36–41).

Another interesting thyroid carcinoma that can present with hyperthyroidism is the rare diffuse follicular variant of papillary carcinoma (42). A tumor that is most often found in young females who present with goiter, the clinical picture resembles classic Graves' disease or toxic goiter. About 25% of these lesions will show hyperthyroidism and abnormal thyroid function tests. Treatment of the cancer will lead to resolution of the metabolic abnormality (40–44).

## Hashitoxicosis

This term originally coined about 40 years ago by the Mayo Clinic group describes patients who present clinically with hyperthyroidism but whose glands show the histology of chronic lymphocytic thyroiditis including oxyphilia (Hürthle cell metaplasia) (45). This histologic presentation is also often seen in children and very young usually teenage patients who present with thyroid hyperfunction. Often these patients go through a phase of euthyroidism and subsequently hypothyroidism over a period of decades (46–48).

## Secondary and tertiary hyperthyroidism

When hyperthyroidism is associated with lesions of the pituitary gland or the hypothalamus, it is considered secondary and tertiary hyperthyroidism respectively. In comparison to primary hyper thyroidism, these clinical conditions are extremely rare (< *1% of hyperthyroidism*). The lesion in the pituitary gland is most frequently multifocal thyrotroph hyperplasia rather than a thyroid stimulating hormone (TSH) producing adenoma. Lesions of the hypothalamus producing thyrotropin releasing hormone (TRH) can stimulate the pituitary thyrotrophes to hyper secrete thyroid stimulating hormone (TSH) and subsequently to influence thyroid gland to produce excess thyroid hormone. Lesions of the hypothalamus responsible for this excess TRH include tumors, granulomatous disease (i.e., sarcoid) and other mass producing lesions (49–53).

## Hyperthyroidism due to struma ovarii

The presence of thyroid tissue within the ovary is usually seen in benign cystic teratomas also known as dermoid cysts of the ovary (54). The thyroid in these lesions is often part of a multi-tissue proliferation, that is tissues from all three embryological germ layers are represented. When thyroid tissue is the only or majority of tissue (>50%) in a teratoma (often termed mono dermal teratoma) it is diagnosed as struma ovarii. In most cases the thyroid either appears normal or shows changes consistent with colloid goiter. In rare instances, the thyroid will appear hyperplastic or even show lymphocytic infiltration mimicking thyroiditis. Rarely neoplasms originating in the thyroid gland including papillary carcinoma, follicular carcinoma or even poorly differentiated carcinoma can arise in a background of struma ovarii. Most of these tumors do not involve the ovarian surface and do not spread (these tumors have been designated by some authors as “proliferating struma”) (55). Unusual situations have been described wherein the struma ovarii or tumors therein may hyper secrete thyroid hormone and lead to clinical hyperthyroidism (56–58).

## Hyperthyroidism associated with ectopic production of thyrotropin (thyroid stimulating hormone- (TSH)) and thyrotropin releasing hormone (TRH)

Rare reported cases of non-endocrine malignant tumors secreting TSH or TRH have been reported. The most common histology is that of hepatocellular carcinoma. The tumor produces these stimulatory hormones and when tumor is entirely removed the levels of hormones drop and hyperthyroidism regresses (59, 60).

## Hyperthyroidism associated with trophoblastic disease

Gestational trophoblastic disease including hydatidiform mole and choriocarcinoma is associated with marked elevation of beta human chorionic gonadotropin (beta HCG). Because the beta subunit of HCG is identical in chemical structure to one of the subunits of TSH, the HCG elevation can mimic elevated TSH and stimulate the thyroid to produce excess thyroid hormone. Although it is rare to see tissue from the thyroid in these patients, it is expected that the gland would show a hyperplastic appearance with papillae and cellular enlargement. Lymphocytic infiltration would be absent. Treatment of the gestational trophoblastic disease by uterine evacuation followed by chemotherapy usually leads to resolution of the hyperthyroid state (61–64).

## Drug associated hyperthyroidism

A variety of classes of pharmaceutical agents can cause thyroid dysfunction. It is beyond the purpose of this review to engage in a lengthy discussion of the clinical disorders caused by these drugs. Some of these interfere with metabolism of iodine, others with the production of thyroid hormone and its conversion to active moieties, and still others do not produce abnormalities in thyroid function but cause chemical interference with thyroid function test measurements. Many drugs can affect thyroid function (phenytoin and derivatives, therapies associated with interleukin administration usually in oncology settings) (65–67). The pathologic counterparts for these include lymphocytic infiltration of the gland with or without fibrosis (68).

Those drugs that cause hyperthyroidism are fewer and they usually exert their effect through interference with the metabolism of iodine. It is rare to see pathological specimens from these patients; the exceptions is the cardiac drug, amiodarone, interleukin containing regimens for chemotherapy and most recently PDL 1 or immune checkpoint inhibitors; these will be discussed below.

### Amiodarone associated thyroid dysfunction (AATD)

The literature notes that there are two types of thyroid lesions that are associated with amiodarone, an iodine containing compound used to treat cardiac arrhythmias. Amiodarone induced thyrotoxicosis (AIT) is classified as type I and type II, the former occurs in patients with underlying thyroid disease such as nodular goiter, autonomous nodular goiter or Graves' disease, whereas, Type II is caused by iodine-led destruction of the thyroid follicular epithelium in a normal thyroid gland. Because amiodarone is vital to control the cardiac problems, it is often not possible to wean the patient from the medication or to change to another drug. The first line of therapy in amiodarone induced thyrotoxicosis is treatment with Thionamides in AIT I and glucocorticoids in AIT II. Thyroid excision is undertaken in patients who do not respond to medical therapy in order to treat the hyperthyroidism which often worsens cardiac symptoms (69–72).

**Figure 5:**
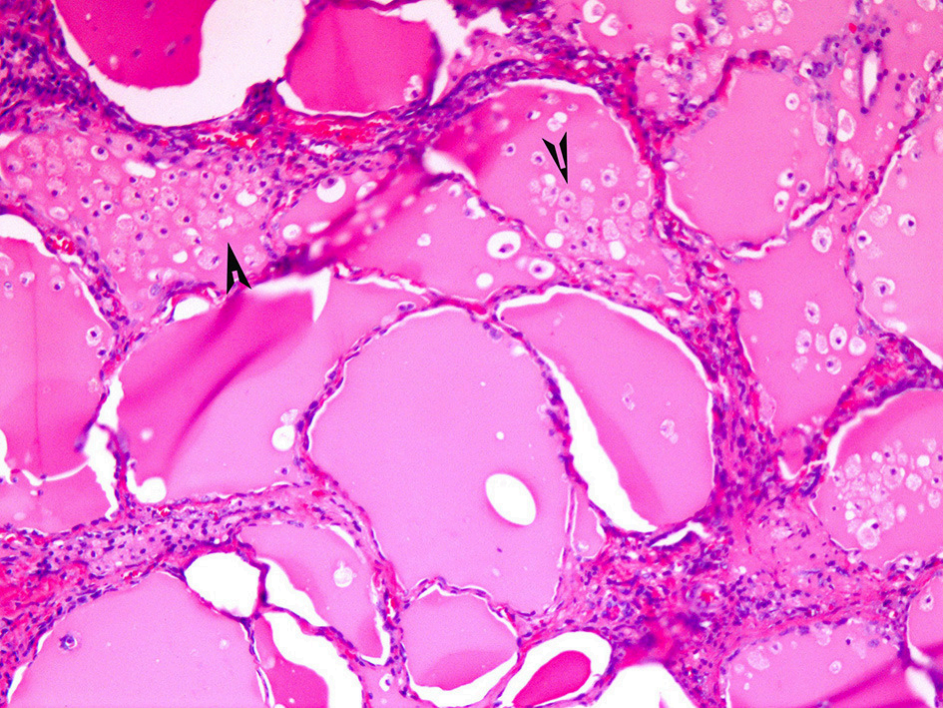
Amiodarone associated follicular cell damage. Low and high power showing large thyroid follicles filled with colloid and numerous histiocytes (arrow heads, 3A,B).

**Figure 6:**
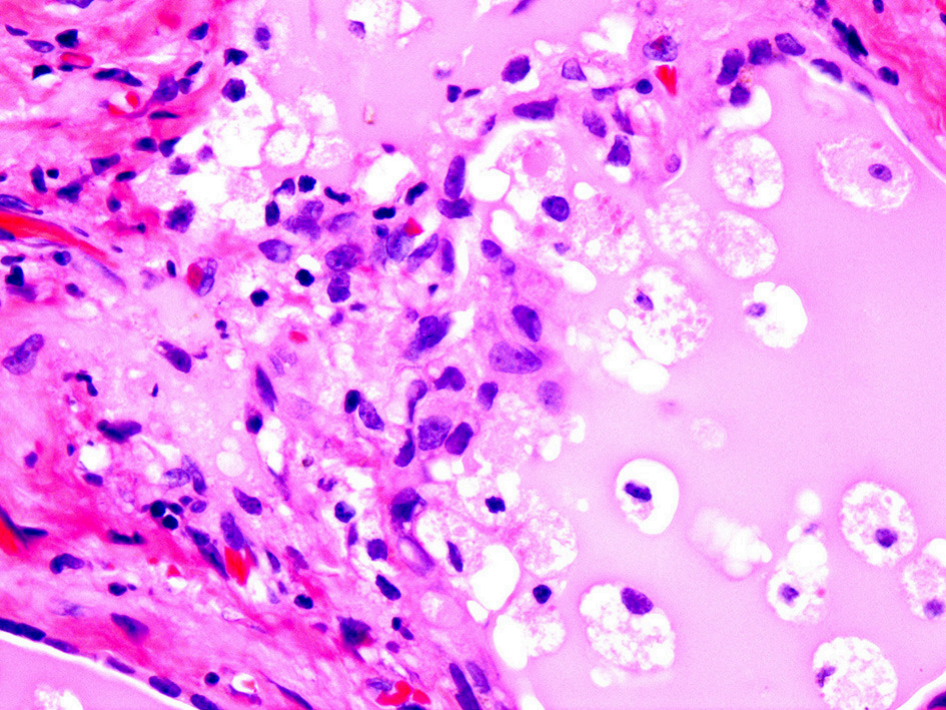
Same as Figure 5.

If the gland is already pathologically abnormal (nodular thyroid goiter, Graves' disease), the pathology of the resected gland shows follicular disruption with histiocytes infiltrating the follicular epithelium and colloid (Figures 5, 6). Rarely, inflammatory cells are noted within the thyroid parenchyma (Type I). On the other hand if the thyroid is histologically normal (Type II), the pathologic lesions show much milder follicular damage (73–75). These changes are similar to those seen in amiodarone induced pulmonary and liver toxicity (76, 77). Ultrastructural studies of both lung and thyroid tissues have shown lysosomal and mitochondrial inclusions in follicular cells consistent with follicle cell destruction (77). However, this simple explanation is not the only reason for the thyroid dysfunction. For example, co-cultures of amiodarone with human thyrocytes have shown the production of interleukin 6 and the drug also decreases the sodium-iodide symporter mRNA in the follicular cells (78).

The removal of the thyroid in amiodarone induced hyperthyroidism results in resolution of the hyperfunction and reversion of the cardiac disorder to baseline (75, 79).

### Hyperthyroidism associated with antineoplastic agents and targeted therapies

Thyroid dysfunction can occur in 20–50% patients receiving antineoplastic agents and targeted therapies. High dose IL-2 therapy can lead to hyperthyroidism in 7% of patients. In rare instances, interferon-alpha treatment can lead to classic Graves' disease and even Graves' opthalmopathy; and these condition can persist even after the cessation of therapy (67, 80). At present, several tyrosine kinase inhibitors (TK1) are being used to treat different types of malignant neoplasms. TKI can lead to various forms of toxicities including those related to endocrine organs. Transient hyperthyroidism can occur during TKI therapy and is often due to destructive thyroiditis (67, 80).

Immune check-point inhibitors with their antitumor activity have shown to improve the survival rates of non-small cell lung carcinoma, melanoma, bladder and renal carcinoma, and ovarian carcinoma. A small number of patients undergoing treatment with immune-checkpoint inhibitors such as anti PD-1/anti-PDL-1 can develop hyperthyroidism (81).

## Mechanico- destructive causes of hyperthyroidism (non-hyperthyroid thyrotoxicosis)

The term mechanico-destructive hyperthyroidism (non-hyperthyroid thyrotoxicosis) has been coined by us to describe those conditions in which relatively rapid destruction of thyroid tissue followed by release of stored thyroid hormone from colloid as follicles or destroyed produces hyperfunction. Both benign and malignant conditions can be associated with this type of hyperthyroidism. An important clinical clue to the possibility of one of these disorders is that in contrast to more common causes of hyperthyroidism, radionucleotide scans show uptakes in the range of 1% or less. This reflects the destruction of the thyroid gland by the inflammatory or neoplastic process; the follicular epithelium is destroyed and cannot take up the radioactive isotope.

### Subacute thyroiditis (“granulomatous thyroiditis”; de quervain thyroiditis)

This condition is believed to be associated with systemic and or thyroid infection usually viral in nature, is often a painful cause of hyperthyroidism. Patients with this disorder will often present with neck pain which may be referred to the jaw or the chest. In the initial phases of this disease symptoms of hyperthyroidism are often clinically evident. As the gland is replaced by the inflammatory granulomatous process, the follicular epithelium is destroyed, follicles of ruptured and stored thyroid hormone within the colloid is released into the circulation. Unlike usual Graves' disease however the thyroid cannot take up iodide and produce more hormone. Thus, a phase of hypothyroidism is noted until healing occurs (82–84).

## Malignant neoplasms causing hyperthyroidism

Malignant neoplasms which are rapidly growing can be associated with this mechanic-destructive type of hyperthyroidism. The tumors most often identified are *anaplastic thyroid carcinoma, malignant lymphoma usually primary in the thyroid and of large cell type and poorly differentiated metastatic cancers* involving the thyroid (breast carcinoma and lung carcinoma most commonly). Histologically one sees the highly malignant tumor freely infiltrating the thyroid, destroying and replacing the tissue, with rupture of the follicles and release of thyroid hormone containing colloid. The rapidity of the process can lead to market elevation of thyroid hormone and a toxic state simulating thyroid storm (85–89).

In affected patients, there is often near complete destruction of the gland and the eventual development of hypothyroidism. Patients need to be supplemented with thyroid hormone to maintain a euthyroid metabolic state; if treatment of the tumor is successful, regeneration of thyroid follicles may occur from the residual thyroid tissue and as in subacute thyroiditis, normalization of thyroid function may occur (86–89).

## Conclusion

This review has described the pathology and clinicopathologic correlations of unusual lesions of the thyroid and extrathyroidal tissues which can show clinical manifestations of hyperthyroidism. Although most of these conditions are rare especially when compared to Graves' disease or toxic nodular goiter, it is important for both the clinician and pathologist to be aware of them as diagnostic considerations.

## Author contributions

VL and ZB have equally contributed to the literature review, drafting the manuscript and obtaining microscopic photographs. Both authors have reviewed the final version of this manuscript before submitting it to topic editors of the journal.

### Conflict of interest statement

The authors declare that the research was conducted in the absence of any commercial or financial relationships that could be construed as a potential conflict of interest.
